# Global research development of chondrosarcoma from 2003 to 2022: a bibliometric analysis

**DOI:** 10.3389/fphar.2024.1431958

**Published:** 2024-08-02

**Authors:** Hansong Duan, Jiatong Li, Jianfei Ma, Ting Chen, He Zhang, Guanning Shang

**Affiliations:** Department of Bone and Soft Tissue Oncology, Shengjing Hospital of China Medical University, Shenyang, China

**Keywords:** chondrosarcoma, bibliometric analysis, cancer, bone tumor, 3D printing

## Abstract

**Background:**

Chondrosarcomas are common primary malignant bone tumors; however, comprehensive bibliometric analysis in this field has not yet been conducted. Therefore, this study aimed to explore the research hotspots and trends in the field of chondrosarcoma through bibliometric analysis to help researchers understand the current status and direction of research in the field.

**Methods:**

Articles and reviews related to chondrosarcoma published between 2003 and 2022 were retrieved from the Web of Science. Countries, institutions, authors, journals, references, and keywords in this field were visualized and analyzed using CtieSpace and VOSviewer software.

**Results:**

Between 2003 and 2022, 4,149 relevant articles were found. The number of articles published on chondrosarcoma has increased significantly annually, mainly from 569 institutions in China and the United States, and 81 in other countries. In total, 904 authors participated in the publication of studies related to chondrosarcomas. Over the past 20 years, articles on chondrosarcoma have been published in 958 academic journals, with *Skeletal Radiology* having the highest number of publications. Furthermore, keywords such as “gene expression,” “radiotherapy,” “experience,” and “apoptosis” have been popular in recent years.

**Conclusion:**

Over the past 20 years, the global trend in chondrosarcoma research has primarily been clinical research, with basic research as a supplement. In the future, communication and exchange between countries and institutions should be strengthened. Further, the future main research hotspots in the field of chondrosarcoma include mutated genes and signaling pathways, precision surgical treatment, proton therapy, radiation therapy, chemotherapy, immunotherapy, and other aspects.

## 1 Introduction

Chondrosarcoma (CS) is a heterogeneous, usually slow-growing primary malignant bone tumor. It is the second most common malignant bone tumor after osteosarcoma, and the formation of transparent cartilage vegetation tissue is a prominent feature thereof (Chow, 2018; Zajac et al., 2021). CSs mainly affect adults and older adults, with men being the most commonly affected. The peak age range for the onset of this disease is 40–70 years. The pelvis and proximal femur are the most common sites of CSs (Tlemsani et al., 2023). Unlike chondromas, CSs contain a large number of plump tumor cells with prominent cell dysplasia. The nucleus is often large or contains binucleated cells, and mitotic figures are relatively rare. Liquefaction, calcification, or ossification may also occur in these tumor cells. CSs grows slowly and invade the surrounding soft tissues. Metastasis is relatively rare and often occurs in the late stages of the disease; however, it usually becomes a highly malignant CS (Whelan and Davis, 2018). The most common sites of metastases are the lungs, bones, and liver, whereas lymph node metastases are relatively rare.

Common prognostic factors for CS include grade, degree of tumor necrosis, and degree of mitosis. Changes in these histological parameters are associated with the risk of recurrence and metastasis. Traditional chemotherapy and radiotherapy are not ideal for treating CSs (Tlemsani et al., 2023), as surgical resection remains the preferred treatment thereof. Most chondrosarcomas exhibit good differentiation; however, incomplete resection can lead to local recurrence. Therefore, in recent years, three-dimensional (3D) printing technology has gradually entered public view. As a popular technology that implements the principle of precision treatment, 3D printing is also a more suitable treatment choice for complex pelvic malignant tumor resection surgery (Chen et al., 2020), as it uses specific materials and digital models to print out structures (Chen et al., 2016; Xu et al., 2016). Since the 1980s, 3D printing technology has been widely used in military, construction, and other fields; in recent years, it has been gradually applied in the medical field (Fan et al., 2015; Liang et al., 2017). 3D printing can create precise personalized prostheses for patients and significantly improve the treatment of various diseases, thereby providing new diagnostic and treatment plans for surgeons. Simultaneously, 3D printing can restore the position of muscle and ligament attachment points on the prosthesis, greatly improving stability and the postoperative functional recovery effect of patients (Wang et al., 2018). In recent years, research on CSs has developed rapidly, both domestically and internationally, and many recent research results have been published regarding disease pathogenesis, pathological types, treatment, and prognosis. However, bibliometric analyses of CSs have not yet been published, and qualitative and quantitative research on their overall quality is relatively limited. Therefore, it is necessary to make a comprehensive prediction and evaluation of the future research focus and development trends in CS so that scholars can have a comprehensive and systematic understanding of the research in this field.

Describing knowledge structures, the evolution of research topics, and the emergence of topics have always been important components of information science (Han et al., 2022). Bibliometrics is an interdisciplinary field that quantitatively analyses all knowledge carriers using mathematical and statistical methods. It is a comprehensive knowledge system that integrates mathematics, statistics, and literature and emphasizes quantification. The measurement objects included a) the contributions of countries/regions, institutions, journals, and authors in the field; b) collaboration between countries, institutions, or authors; c) distribution of journals; and d) knowledge base (Miao et al., 2022). Utilizing visual information technology tools and methods, the development process, current status, and trends in research content can be displayed visually. With the continuous improvement of academic levels in various countries worldwide, the number of existing studies in different disciplines is large. Traditional review articles find it difficult to summarize the overall development trends and research hotspots of a certain discipline, whereas bibliometric analyses rely on the experience and knowledge of researchers to treat science as a knowledge-generation system (Luo et al., 2021; Zhang et al., 2022).

This study aims to analyze the changes in research hotspots in the field of CS from 2003 to 2022 and examine future global development trends through bibliometric analysis. We hope that our study highlights the main research directions and provides new methods and ideas for future research on CSs.

## 2 Materials and methods

### 2.1 Data sources

We collected publishing information from the Web of Science (SCIExpanded), which has been considered the best database for bibliometrics (Aggarwal et al., 2016).

### 2.2 Literature retrieval strategy and data collection

This retrospective study evaluated data publicly available online and in libraries and, therefore, did not require approval from institutional review committees (Zhang et al., 2022). We conducted a comprehensive search of relevant publications using the Web of Science (WoS) Core Collection Science Citation Index Expanded (SCIExpanded) database on 27 February 2023. The search terms were as follows: Topic = Chondrosarcoma and Document Type= (Article or Review) (Available Publications = 4,841). Publications in languages other than English (available publications = 4,637) and those published outside of 1 January 2003 to 31 December 2022 (available publications = 4,149) were excluded. All records and references were exported in plain text and tab-separated file formats. In addition, these files were named “download_. Txt.” The txt format data obtained from the Web of Science were exported to CiteSpace [version 6.1] R6 (64-bit), and VOSviewer (version 1.6.19), as well as the bibliometric online analysis platform (http://bibliometric. com/), were used for the subsequent bibliometric analyses. The inclusion criteria for the literature were as follows: 1) the manuscript focuses on the treatment of CS with complete content, 2) the types of literature are articles and reviews, and 3) the article is written in English. The exclusion criteria include: 1) the topic is not related to CS or not suitable for evaluation and 2) the article includes conference abstracts, news, and briefings.

### 2.3 Bibliometric analysis

We used CiteSpace [version 6.1] R6 (64-bit) and VOSviewer (version 1.6.19) to analyze the statistical data and visualize a scientific knowledge graph. Knowledge graphs can help researchers intuitively understand research hotspots and their evolutionary processes and predict research development trends in areas of interest (Ma et al., 2021). In addition, the annual publication quantities and growth trends of different countries/regions were determined through online bibliometric platforms.

CiteSpace [version 6.1. R6 (64 bit)] is a software developed by Professor Chaomei Chen for visualizing and analyzing massive amounts of literature. It can analyze the themes, keywords, author affiliations, and collaborative networks of literature in databases such as WoS. Visual analysis of journals, publication dates, and citations of literature can help researchers quickly understand the development process of a certain field, identify key literature and main research teams in that field, and elucidate research frontiers and development trends in that field.

VOSviewer is a free JAVA-based bibliometric mapping software developed by Van Eck and Waltman in 2009. This software can conduct analyses regarding the distribution structure, quantity structure, and change pattern of the literature, as well as their titles, keywords, word frequencies, and citation information. It is suitable for analyzing scientific development dynamics, scientific research overviews, and disciplinary development trends; furthermore, it places more emphasis on the visualization of scientific knowledge. In addition, VOSviewer has a powerful ability to process and display large-scale bibliometric graphs in an easy-to-understand manner (van Eck and Waltman, 2010).

## 3 Results

### 3.1 Annual circulation trend of publications

**FIGURE 1:**
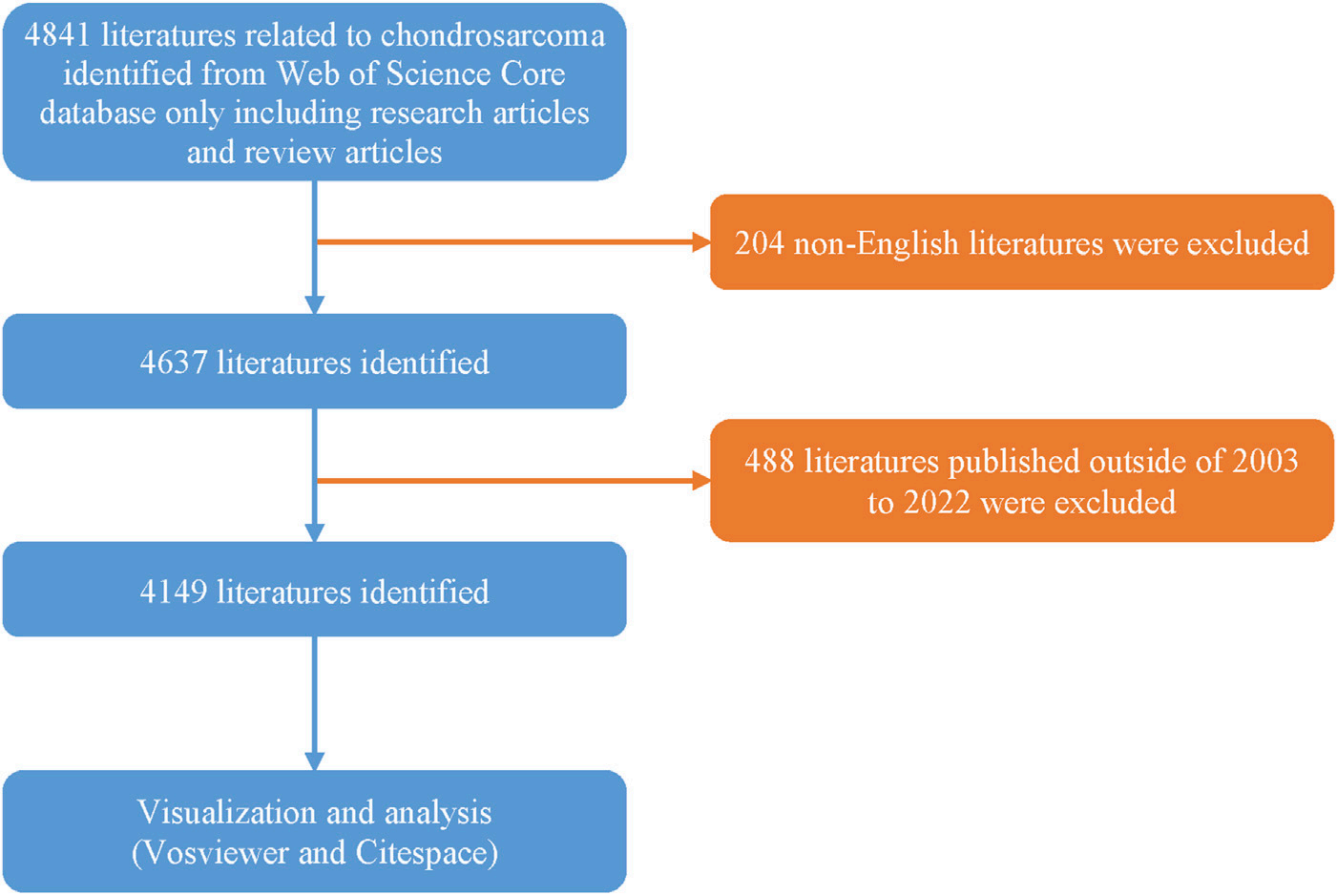
Flow diagram of the literature screening and inclusion process.

**FIGURE 2:**
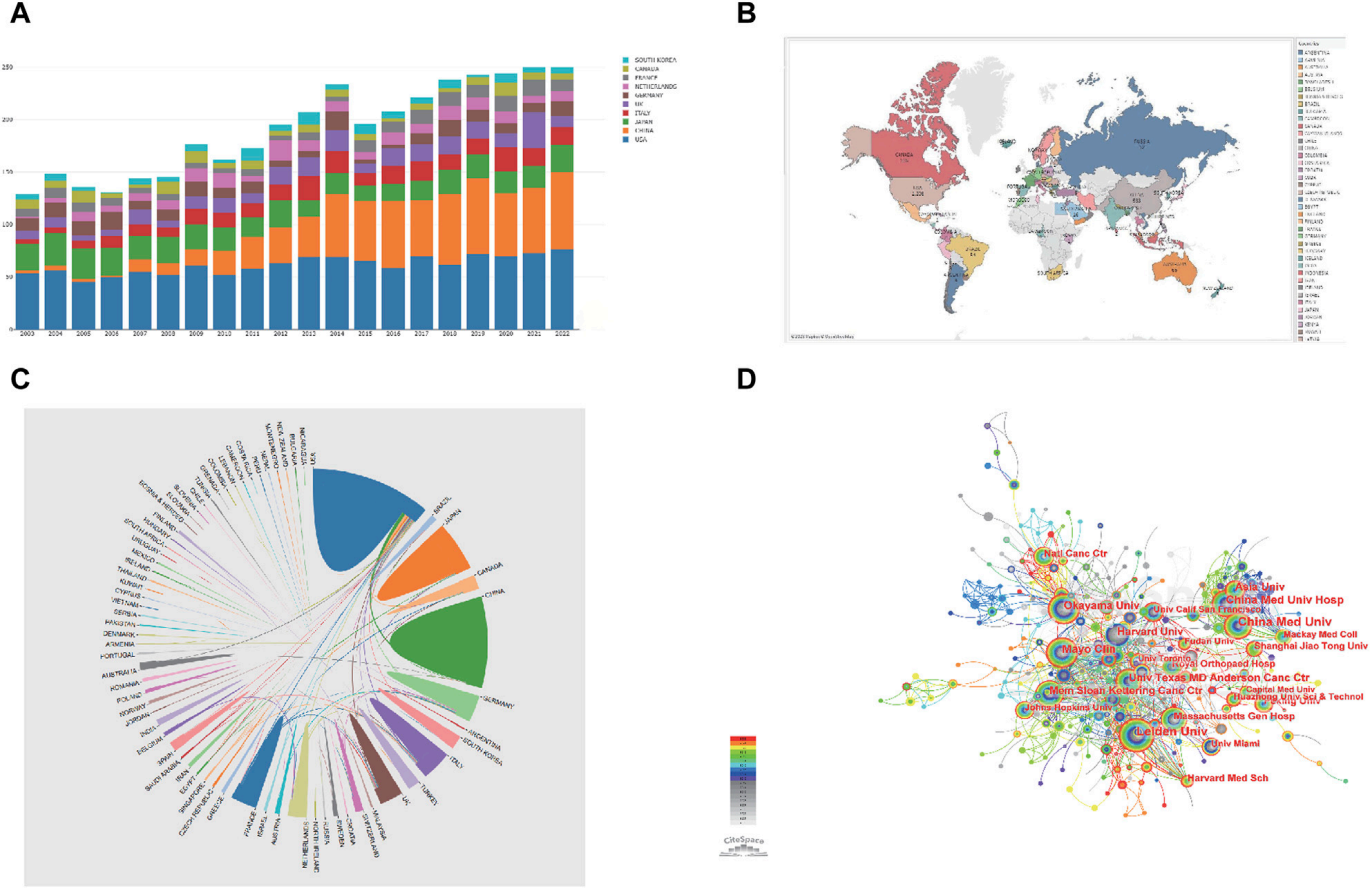
Collaboration between countries/regions and institutions from 2003 to 2022. **(A)** Top 10 countries/regions with Chondrosarcoma research in publication rankings from 2003 to 2022. Different colors represent different countries. **(B)** A world map displaying the number of articles published on chondrosarcoma in different countries. **(C)** Cooperation among various countries in the field of chondrosarcoma from 2003 to 2022. **(D)** CiteSpace visualization map of institutions involved in chondrosarcoma. Each concentric circle represents an institution, and the larger the circle, the more publications produced by that institution. The width of different colored bands in concentric circles represents the circulation of publications produced by different institutions in different years. The lines between each circle represent cooperation and exchange between institutions, and the wider the lines, the closer the cooperation.

From 2003 to 2022, 4,149 papers met the inclusion criteria (Figure 1). As shown in Figure 2A, during the first decade (2003–2012), the global annual publication volume of literature on CS was less than 200 articles, whereas, during the middle 5 years (2013–2017), the annual publication volume increased and remained stable between 200 and 250 articles. In the past 5 years (2018–2022), the publication volume has remained stable at approximately 250 articles with a slow growth trend. Overall, from 2003 to 2022, the number of publications on CS steadily increased every year.

### 3.2 Distribution characteristics of countries/regions and institutions

A total of 569 institutions from 83 different countries/regions have contributed to the field of CSs. Figure 2B shows that the top five countries regarding publication output are the United States (n = 1,208, accounting for 29.1% of the total), China (n = 583, 14%), Japan (n = 445,10.7%), Italy (n = 276, 6.7%), and the United Kingdom (n = 243, 5.9%) (Table 1). The influence of Canada and the United States is very prominent, with centralities of 0.25 and 0.23, respectively. Although China ranks second worldwide regarding publication quantity, its centrality is low, indicating that only a few high-quality papers have been published. Overall, the United States has an absolute advantage in publishing, collaborating, and researching CS and is currently in a leading global position in this research field. However, China and Japan’s annual publication volume in this field lags far behind that of the United States (Figure 2B).

**TABLE 1:** The top 10 countries/regions and institutions related to chondrosarcoma.

Rank	Countries	Count	Centrality	Institutions	Count	Centrality
1	United States	1,208	0.23	China Med Univ (China)	123	0.03
2	China	583	0	Leiden Univ (Netherland)	119	0.11
3	Japan	445	0.04	China Med Univ Hosp (China)	87	0.08
4	Italy	276	0.12	Mayo Clin (United States)	79	0.25
5	England	243	0.16	Okayama Univ (Japan)	68	0.13
6	Germany	215	0.08	Univ Texas MD Anderson Canc Ctr (United States)	65	0.04
7	Netherlands	188	0.01	Asia Univ (China)	64	0.01
8	France	175	0.04	Harvard Univ (United States)	57	0.04
9	Canada	136	0.25	Mem Sloan Kettering Canc Ctr (United States)	50	0.19
10	South Korea	124	0.04	Peking Univ (China)	46	0.01

As shown in Figure 2C, the connection lines between countries are relatively sparse, indicating that there is little cooperation between countries. Some countries, such as the United Kingdom, Italy, the Netherlands, France, Japan, and the United States, cooperate relatively closely. The United States has the closest cooperation with Asian countries, such as China and Japan, followed by Western European countries, such as France and Germany.

The institutions with the most published papers are China Medical University (n = 123, 3%), followed by Leiden University (n = 119, 2.9%), China Medical University Hospital (n = 87, 2.1%), Mayo Clinic (n = 79, 1.9%), and Okayama University (n = 68, 1.6%) (Table 1). Among the top 10 institutions, China and the United States account for 40% respectively, while the remaining 20% are in the Netherlands and Japan. The connections between various institutions are relatively close, and academic exchanges between institutions such as Mayo Clinic, Okayama University, China Medical University, and Harvard University are more frequent (Figure 2D).

### 3.3 Distribution characteristics of authors and co-cited authors

**FIGURE 3:**
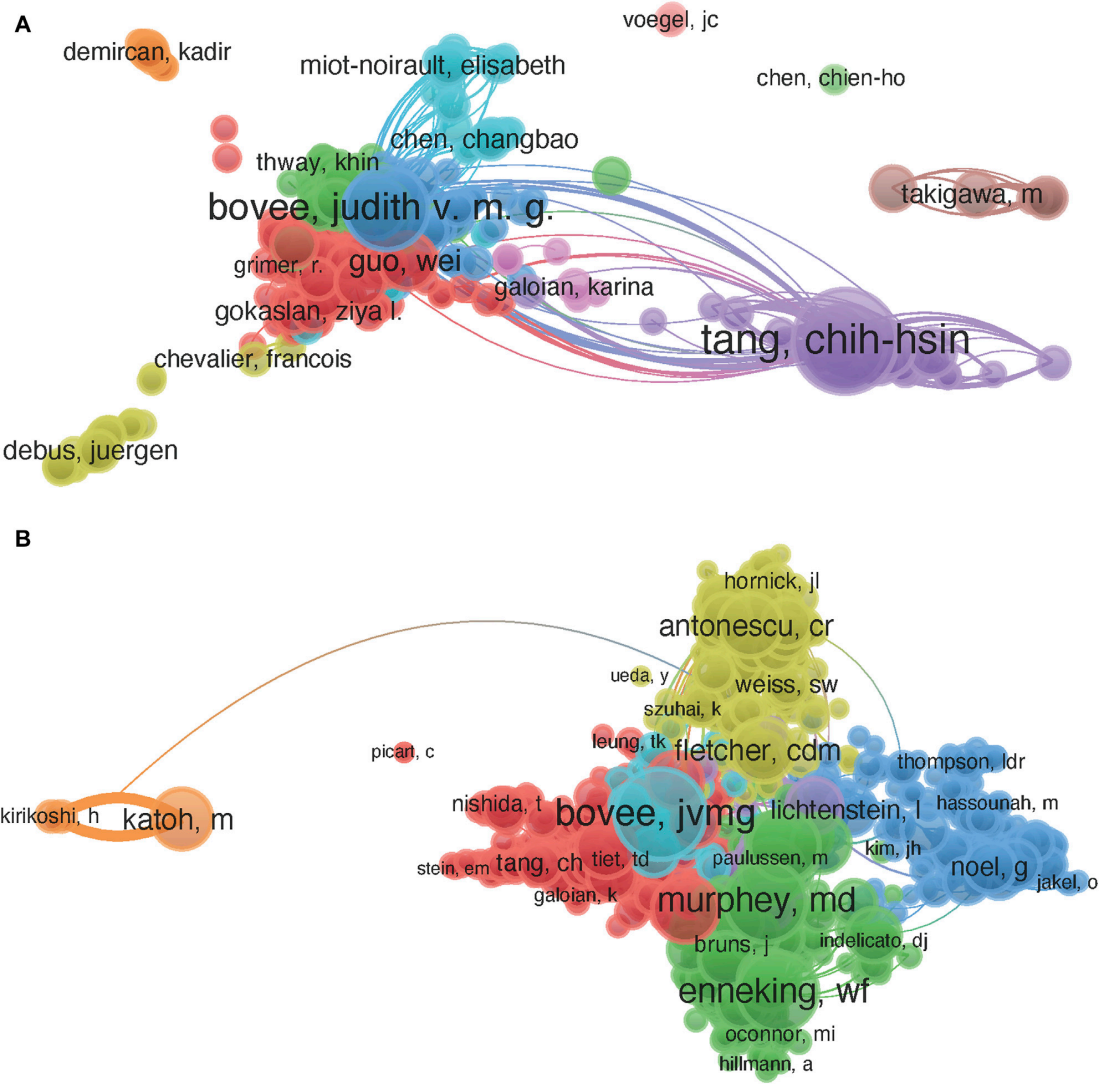
Collaboration between authors from 2003 to 2022. **(A)** Mapping of the authors related to chondrosarcoma. **(B)** Mapping of the co-cited authors related in chondrosarcoma research.

In total, 904 authors participated in the publication of studies related to CSs. As shown in Table 2, Tang (n = 96) has published the most papers, followed by Bovee (n = 61), Fong (n = 56), Hogendoorn (n = 29), and Guo (n = 27). Among the top 10 authors regarding publication volume, we can see that the majority of authors have low centrality. Bovee and Tang have both had a high level of influence in the field of CS and have collaborated more frequently with other authors (Figure 3A).

**TABLE 2:** The top 10 authors and co-cited authors of chondrosarcoma research.

Rank	Author	Count	Centrality	Year	Co-cited author	Citation	centrality
1	Tang CH	96	0.01	2008	Bovee JVMG	375	0.07
2	Bovee JVMG	61	0.03	2007	Harry L evans MD	339	0.02
3	Fong YC	56	0.01	2008	Gelderblom H	312	0.02
4	P.C.W. Hogendoorn	276	0.01	2007	Murphey MD	307	0.02
5	Guo W	27	0.01	2010	Enneking WF	299	0.03
6	Wang SW	25	0.01	2014	Dahlin DC	233	0.05
7	Francis J Hornicek	22	0.01	2007	Unni KK	222	0.01
8	Lin CY	19	0.01	2013	Fletcher CDM	221	0.03
9	Takigawa M	18	0.01	2003	Lee FY	210	0.02
10	Liu JF	14	0.01	2010	Ozaki T	202	0.03

As shown in Figure 3B, the top 10 co-cited authors were cited more than 200 times. The most commonly cited authors are Bovee (n = 375), followed by Evans (n = 339), Gelderblom (n = 312), Murphey (n = 307), and Enneking (n = 299).

### 3.4 Journals and co-cited journals

Articles on CS have been published in 958 academic journals over the past 20 years. As shown in Table 3, the journal with the highest publication volume is *Skeletal Radiology* (n = 78), followed by *Clinical Orthopedics and Related Research* (n = 77), *World Neurosurgery* (n = 45), *Oncology Letters* (n = 38), and *American Journal of Surgical Pathology* (n = 37). Among the top 10 journals regarding literature circulation, *Modern Pathology* has the highest impact factor, with five journals having a JCR partition of Q1.

**TABLE 3:** The top 10 journals and co-cited journals involved in chondrosarcoma.

Rank	Journal	Count	If (2023)	JCR (2023)	Co-cited journal	Citation	If (2023)	JCR (2023)
1	Skeletal Radiology	78	1.900	Q3	Clinical Orthopaedics and Related Research	3,650	4.199	Q1
2	Clinical Orthopaedics and Related Research	77	4.199	Q1	Cancer	3,210	6.100	Q1
3	World Neurosurgery	45	1.900	Q3	Journal of Bone and Joint Surgery-American Volume	3,055	4.399	Q1
4	Oncology Letters	38	2.500	Q3	American Journal of Surgical Pathology	2,963	4.500	Q1
5	American Journal of Surgical Pathology	37	4.500	Q1	Journal of Biological Chemistry	2,747	4.000	Q2
6	Cancer	36	6.100	Q1	Cancer Research	2,115	12.500	Q1
7	Human Pathology	36	2.700	Q2	Journal of Clinical Oncology	2091	42.101	Q1
8	Modern Pathology	36	7.100	Q1	Skeletal Radiology	1879	1.900	Q3
9	Virchows Archiv	36	3.400	Q1	International Journal of Radiation Oncology Biology Physics	1873	6.399	Q1
10	Journal of Surgical Oncology	35	1.999	Q3	Journal of Neurosurgery	1,591	3.500	Q1

**FIGURE 4:**
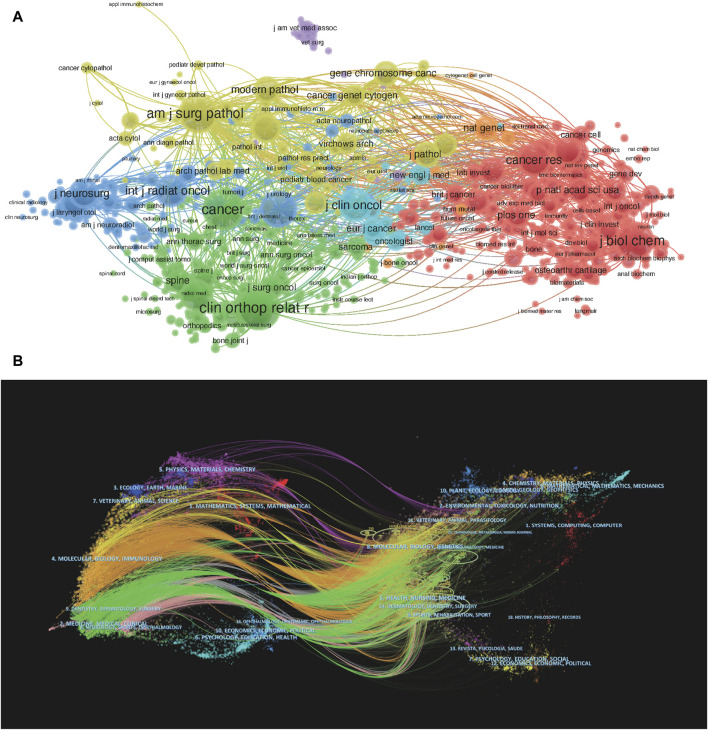
Collaboration between journals from 2003 to 2022. **(A)** Mapping of the co-cited journals related to this field. The size of a point represents the frequency of referencing. The lines between different points indicate that they are cited in a paper. The shorter this line, the closer the connection between the two papers. Dots of the same color indicate that they belong to the same research field. **(B)** The dual map overlay of journals on chondrosarcoma. The citing journals are located on the left, and the cited journals are located on the right. The color paths (orange and green reference paths) represent the cited relationship.

The journal with the highest co-citation frequency is *Clinical Orthopedics and Related Research*, with a co-citation frequency of 3,650 (Table 3), followed by *Cancer* (n = 3,210), *Journal of Bone and Join Surgery-American Volume* (n = 3,055), *American Journal of Surgical Pathology* (n = 2,963), and *Journal of Biological Chemistry* (n = 2,747). The *Journal of Clinical Oncology* far surpasses other journals with an IF of 42.101. Except for *Skeletal Radiology* and *Journal of Biological Chemistry*, the top 10 co-cited journals are all located in the Q1 region (Figure 4A).

The colored flow bands in Figure 4B represent the connections between different journals and cited journals. The left side represents the main journal topic distribution of literature related to CS, whereas the right side represents the main cited journal topic distribution. The results indicate that journals with themes such as “medicine, medical, clinical” and “molecular, biology, immunology” are mainly cited by journals with themes such as “molecular, biology, genetics” and “health, nursing, medicine.”

### 3.5 Co-cited references and reference citation explosion

**FIGURE 5:**
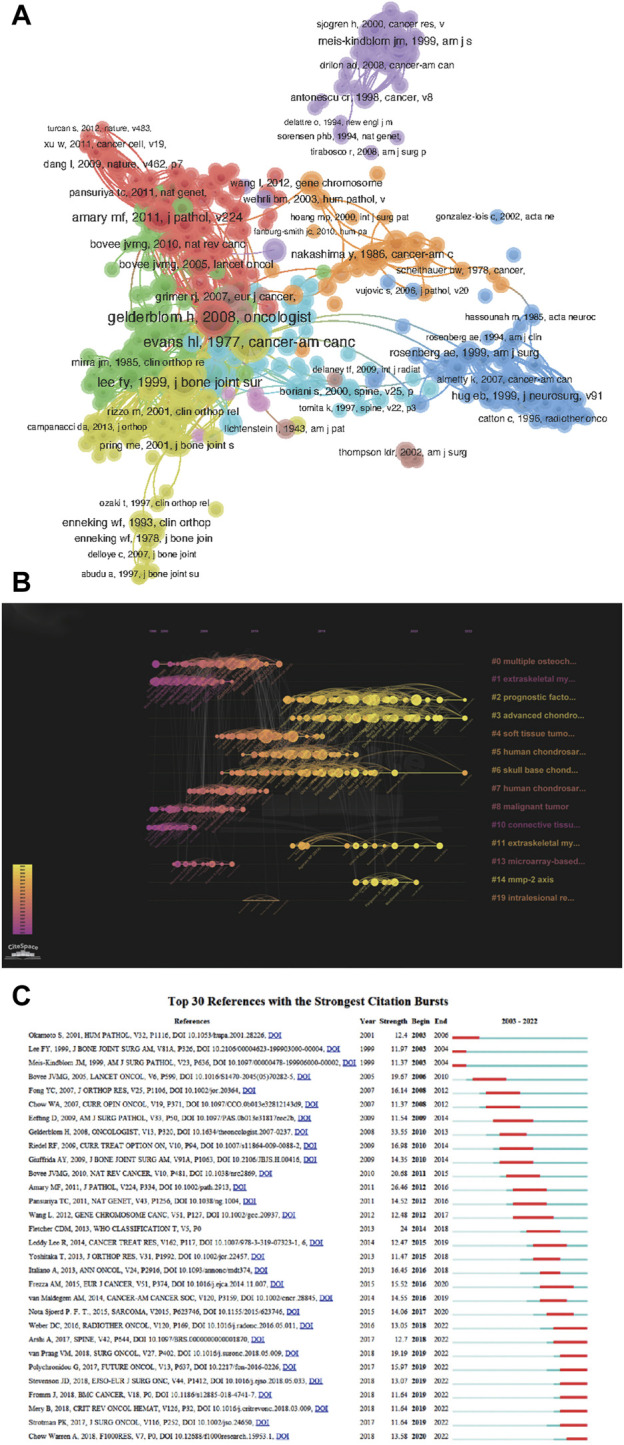
**(A)** Mapping of the co-cited references related to chondrosarcoma. **(B)** Timeline visualization map of co-cited references in the field of chondrosarcoma research. **(C)** CiteSpace visualization map of top 30 references with the strongest citation bursts involved in chondrosarcoma.

Among the 1,256 cited references, the top two were cited more than 50 times regarding frequency, and the top-ranked reference, *The Clinical Approach Towards Chondrosarcoma*, was written by Gelderblom as the first author (Table 4). Figure 5A shows a visual network graph of cited references in the field of CS research over the past two decades. From the graph, we can see that there is a strong correlation between the references, and most of the highly cited references have been published in the past decade.

**TABLE 4:** The top 10 co-cited references related to the chondrosarcoma.

Rank	Year	Author	Title	Journal	Citation	Centrality
1	2008	Gelderblom H	The clinical approach towards chondrosarcoma	Oncologist	74	0.03
2	2011	Amary MF	IDH1 and IDH2 mutations are frequent events in central chondrosarcoma and central and periosteal chondromas but not in other mesenchymal tumours	Journal of Pathology	63	0.05
3	2010	Bovee JVMG	Cartilage tumors and bone development: molecular pathology and possible therapeutic targets	Nature Reviews Cancer	47	0.1
4	2018	van Praag VM	Incidence, outcomes and prognostic factors during 25 years of treatment of chondrosarcomas	Surgical Oncology-Oxford	46	0.02
5	2017	Polychronidou G	Novel therapeutic approaches in chondrosarcoma	Future Oncology	45	0.03
6	2009	Riedel RF	The clinical management of chondrosarcoma	Current Treatment Options in Oncology	39	0.01
7	2015	Frezza AM	Mesenchymal chondrosarcoma: Prognostic factors and outcome in 113 patients. A European Musculoskeletal Oncology Society study	European Journal of Cancer	38	0.03
8	2005	Bovee JVMG	Emerging pathways in the development of chondrosarcoma of bone and implications for targeted treatment	Lancet Oncology	38	0.02
9	2013	Italiano A	Advanced chondrosarcomas: role of chemotherapy and survival	Annals of Oncology	37	0.01
10	2017	Arshi A	Chondrosarcoma of the Osseous Spine	Spine	36	0.02

To explore the changes in research hotspots over time, we conducted a further cluster analysis of the co-cited literature on CS. Figure 5B shows a timeline visualization map of cited references in the field of CS research, comprising 857 nodes and 3,935 links. The frequency of the links in the graph determines the degree of connection tightness. According to the timeline, the four themes of “multiple osteochondroma” (Cluster 0), “extraskeletal myxoid chondrosarcoma” (Cluster 1), “integrated expression” (Cluster 7), and “malicious tutor” (Cluster 8) were relatively popular focal points in the early 21st century. The three themes of “soft tissue tumor” (Cluster 4), “human chondrosarcoma cell” (Cluster 5), and “skull base chondrosarcoma” (Cluster 6) were the focus of research during the decade from 2007 to 2017, indicating that research in this decade has focused extensively on the histology and pathophysiology of CS. The two thematic clusters of “diagnostic factor” (Cluster 2) and “advanced chondrosarcoma” (Cluster 3) have shown more activity in the past decade’s timeline, indicating that current and future research trends are increasingly focused on the prognostic factors of CS and the progression of tumor treatment.

Figure 5C shows the 30 strongest reference citation outbreaks in the field of CS over the past two decades. The graph shows that the first citation outbreak occurred in 2003 and most of the co-cited references have been frequently cited in the past 15 years.

### 3.6 Keyword and hotspot analysis

**FIGURE 6:**
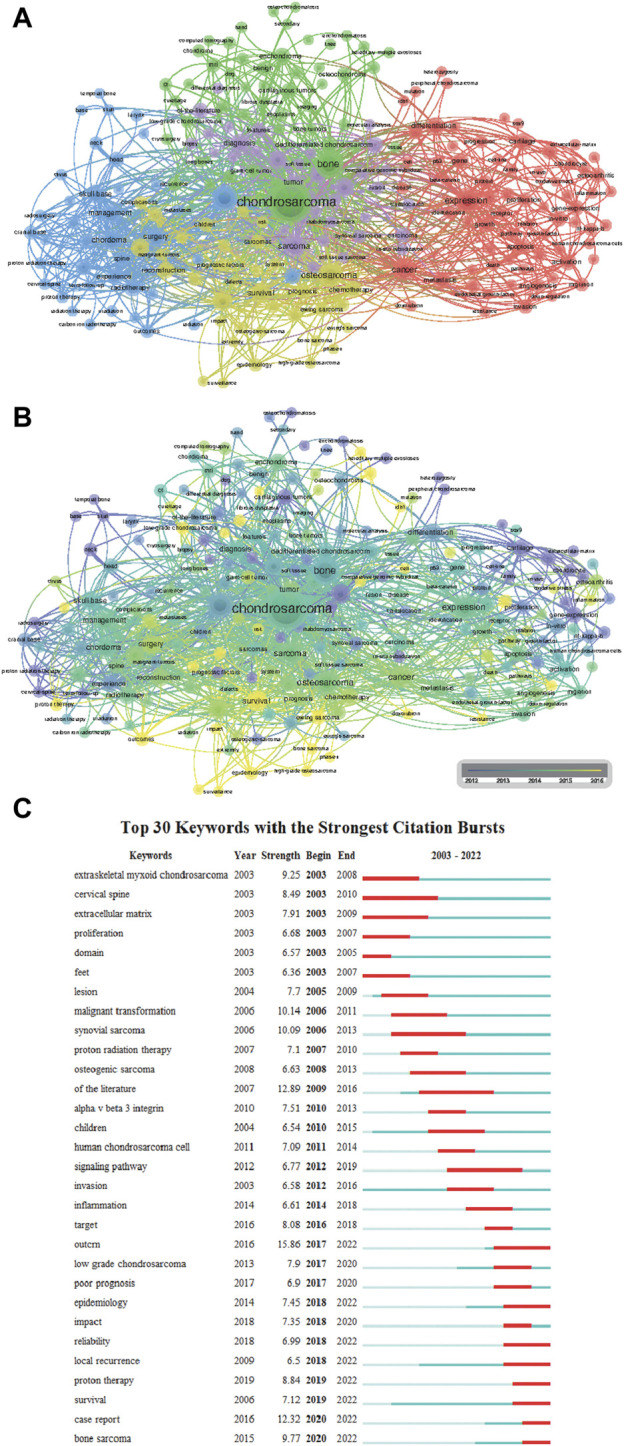
Visualization of co-occurrence analysis based on chondrosarcoma from 2003 to 2022. **(A)** Mapping of keywords in the research on chondrosarcoma from 2003 to 2022; the frequency is represented by the size of points. **(B)** Distribution of keywords based on average frequency of occurrence. **(C)** CiteSpace visualization map of top 25 keywords with the strongest citation bursts involved in chondrosarcoma.

We have identified the top 20 keywords with the highest co-occurrence frequency (Table 5), among which the most common keyword is “chondrosarcoma” (n = 626), and those with co-occurrence frequencies exceeding 200 include “bone” (n = 593), “tumor” (n = 571), “expression” (n = 456), “diagnostic factor” (n = 277), “cancer” (n = 270), “osteosarcoma” (n = 260), “sarcoma” (n = 227), “management” (n = 206), and “skill base” (n = 200). Figure 6A shows a co-occurrence network graph of these keywords (with one keyword appearing ≥25 times). From the graph, it can be seen that key nodes such as “gene expression,” “radiotherapy,” “experience,” and “apoptosis” have been popular in recent years, indicating that the current research trend in CS is more inclined towards genetic and cellular morphological aspects as well as treatment plans (Figure 6B).

**TABLE 5:** The top 20 keywords related to chondrosarcoma.

Rank	Keyword	Count	Rank	Keyword	Count
1	Chondrosarcoma	626	11	Survival	195
2	Bone	593	12	Extraskeletal myxoid chondrosarcoma	185
3	Tumor	571	13	Differentiation	171
4	Expression	456	14	Resection	166
5	Prognostic factor	277	15	Mesenchymal chondrosarcoma	162
6	Cancer	270	16	Bone tumor	160
7	Osteosarcoma	260	17	Chordoma	158
8	Sarcoma	227	18	Soft tissue	152
9	Management	206	19	Diagnosis	150
10	Skull base	200	20	Gene	149

The top 30 keywords with strong citation bursts in the papers on CS are presented in Figure 6C. Four keywords have been cited for the longest duration of the outbreak, all lasting for 7 years, namely, “cervical spine” (2003–2010), “synovial sarcoma” (2006–2013), “of the literature” (2009–2016), and “signaling path” (2012–2019).

## 4 Discussion

### 4.1 General information

At present, bibliometric analysis is widely applied in the medical field to evaluate future research trends and hotspots in a certain medical field, so that future research teams and doctors can explore more disease mechanisms and treatment methods. However, there are currently no articles published regarding the bibliometric analysis of CSs. In this study, we conducted a scientific search of English papers related to CS in the WoS database from January 2003 to December 2022 and retrieved 4,149 articles for bibliometric analysis. Through targeted statistics and analysis of articles related to CS over the past 20 years, we can roughly understand the future development trends and directions in this field.

From Figures 2A, B, we can see that the annual growth trend of global publications related to CS is generally increasing. From 2003 to 2008, the number of papers published each year was less than 150, indicating that research on CSs is still in its early stages of development. From 2009 to 2014, the publication volume of CS-related papers showed a stable growth trend. We can see that there was an explosive growth in the number of publications between 2009 and 2014. The surge in publications in 2009 may be closely related to *The Clinical Approach Towards Chondrosarcoma*, which was published by Gelderblom in 2008. This article mainly focuses on the histopathology, classification, diagnostic methods, and treatment of CS and has had an extremely profound impact on subsequent research teams researching CS. From 2013 to 2014, the annual circulation volume experienced a brief explosive growth, which may be related to China’s breakthrough progress in CS research. From 2015 to 2022, the global annual publication volume maintained a slow growth trend, indicating that research on CS has gradually entered the public eye and received widespread attention from scholars. Overall, the average annual publication volumes in various countries remained stable over the past two decades. China’s publication volume was less than 25 articles from 2003 to 2007, but from 2009 to 2014, China’s publication volume showed an increasing trend annually and remained stable at approximately 50 articles after 2015. This indicates that China’s attention and research on CSs increased between 2009 and 2014.

Centrality is used to measure the importance of the nodes in a network. The higher the node’s centrality, the greater its role in the network. The United States has the highest centrality, followed by the United Kingdom and Canada, indicating that these three countries are in an international core position in CS research. Among all the top 10 countries regarding publication volume, all are developed countries, except for China. Among the top 10 institutions regarding publication volume, the United States and China each have 4, indicating that the academic level of institutions in these two countries is at the forefront of the world regarding CS research. Furthermore, although China ranks second in the world in terms of publication quantity, its centrality is very low; therefore, its academic influence level and cooperation and exchange with other countries in the past two decades must be strengthened. In addition, academic exchanges between institutions within the same country are more frequent, while there are fewer connections between countries, indicating that research in the field of CS has more cooperation within countries, and international cooperation must be further improved. In the future CS research, international cooperation, and exchange will become an important part of the development trend.

From the perspective of authors and co-cited authors, although Tang has published the most papers but received relatively few citations, Bovee ranks second in the world regarding publication volume and first regarding co-citation and centrality. Therefore, Bovee holds a high academic position in CS research. In addition, the centrality of the top 10 authors with the highest number of published papers and the top 10 co-cited authors is not high, indicating a lack of cooperation among the authors. Future academic research should aim to strengthen communication and exchange.

Among the top 10 journals regarding publication volume, there are five journals related to oncology and pathology: *Oncology Letters*, *American Journal of Surgical Pathology*, *Human Pathology*, *Modern Pathology*, and *Journal of Surgical Oncology*. Therefore, research on the pathology and oncology of CSs is currently a popular topic and a future research direction.

Among the top 10 cited articles, six are related to the treatment of CS, mostly regarding chemotherapy and drug therapy. The top-ranked reference, *The Clinical Approach Towards Chondrosarcoma*, can be regarded as the cornerstone of CS research, opening new paths for subsequent scholars to conduct scientific research. According to the top 30 papers with the highest citation explosion rate, the majority of reference citations in the past 20 years have lasted for about 3–5 years, indicating the possibility of further explosive progress in future research related to CS. Therefore, it can also be observed that research hotspots change constantly with time and research progress.

In the past two decades, the research hotspots in the field of CS have gradually shifted from the epigenetic and morphological aspects of the disease to deeper molecular-level research. In addition, in recent years, the successive citation outbreaks of keywords such as “local recurrence” (2018–2022), “proton therapy” (2019–2022), and “survival” (2019–2022) indicate that future research in the field of CS will be more focused on disease prognosis and treatment methods.

### 4.2 Hotspots and frontiers

Keywords serve as the main research content and direction of literature and as academic symbols and information carriers after summarizing the literature. By analyzing keywords, we can learn about the latest cutting-edge developments in the field of CSs. According to Figure 6, we can comprehensively analyze that in the past two decades, the six hot keywords “gene targets” (2016–2018), “signaling pathways” (2012–2019), “immunotherapy” (2012–2015), “proton therapy” (2019–2022), “chemotherapy” (2014–2016), and “surgical treatment” (2014–2016) have played a crucial role in promoting the progress of chondrosarcoma treatment in their respective high-frequency years. In the past 20 years, the frequency of these six keywords appearing each year has been increasing overall, indicating that researchers’ attention to these hot keywords has also been increasing year by year. At the same time, it indirectly reflects that the publications published on these six hot topics in the past 20 years have also been increasing year by year. Based on the high-frequency keywords mentioned above, it is clear that current research on CS mainly focuses on genetics (mutated genes and signaling pathways) and clinical treatment (immunotherapy, proton therapy, chemotherapy, surgical treatment).

#### 4.2.1 Genetic mutations in CS

The efficacy of traditional anti-cancer drugs in combating CS has gradually declined and researchers have been trying to identify new therapeutic targets in recent years (Vuong et al., 2021). By studying gene mutations in CSs, researchers can develop more effective drug treatments. According to published research findings, common gene loci in CS include the isocitrate dehydrogenase 1/2 (IDH1/2) gene, type II collagenα 1 chain (COL2A1), and TP53 (Tarpey et al., 2013; Vuong et al., 2021).

The IDH1/2 gene is the most common mutation site in CS. IDH1 and IDH2 are crucial NADP + dependent enzymes in the tricarboxylic acid cycle, responsible for catalyzing the oxidative decarboxylation of isocitrateα, ketoglutaric acid (α-KG) plays a promoting role in glucose metabolism. IDH1 and IDH2 are present in the cytoplasm and mitochondria of cells, respectively (Amary et al., 2011). IDH1/2 mutations can be detected in approximately 50% of central CS and 60% of undifferentiated CS (Amary et al., 2011; Schaap et al., 2013). Mutations in these genes result in ineffective conversion of isocitrate toα, and KG reduces its activity, leading to the accumulation of the metabolite 2-hydroxyglutarate (2HG). Simultaneously, increases in the levels of 2HG also cause a series of epigenetic changes that inhibit the demethylation process of DNA and histones, ultimately leading to high methylation of DNA and histones (Dang et al., 2009). IDH mutations are widely present in endogenous chondromas and CSs and are considered early events in the occurrence and development of cartilaginous tumors. An IDH1 inhibitor, Ivosidenib (AG-120), has been approved for treating relapsed or refractory acute myeloid leukemia (Tlemsani et al., 2023). In the near future, we believe that IDH1 inhibitors, as well as IDH1 and IDH2 combination inhibitors, will be developed and applied for the treatment of CS (Tlemsani et al., 2023).

Tarpey et al. have found mutations in the cartilage collagen gene COL2A1, including gene rearrangement, deletion, and insertion, in 37% of CS (Tarpey et al., 2013). COL2A1 gene mutations are mainly present in central CSs, as well as in some undifferentiated and clear cell CSs (Aigner et al., 1996). The main collagen component of articular cartilage is type II collagen fibers, where α chain is encoded by COL2A1. The occurrence of CS may be related to the impact of COL2A1 mutation on its normal differentiation process, leading to the deposition of the extracellular matrix and limited signal transduction function.

Mutations in TP53 are the most common in human tumors (Tarpey et al., 2013; Nazeri et al., 2018). Josephine et al. have conducted a comprehensive genomic analysis of a portion of dedifferentiated CS cases. Compared to conventional CSs, tumor mutations are more frequent in dedifferentiated CSs, and the frequency of TP53 promoter mutations is significantly increased (Dermawan et al., 2023). This indirectly confirmed the correlation between TP53 overexpression and higher histological grading of CS. Loss of this gene function plays a role in the progression and deterioration of CS (Asp et al., 2001; Sandberg, 2004; Bovee et al., 2010).

#### 4.2.2 Signal pathways related to CS

##### 4.2.2.1 PI3K-AKT mTOR pathway

The mammalian target of rapamycin (mTOR) is a serine/threonine tyrosine kinase that serves as a regulatory factor in the mTOR pathway and plays a crucial role in cell proliferation, metabolism, development, and angiogenesis (Zhang et al., 2013; Conciatori et al., 2018). The PI3K-AKT signaling network is responsible for the direct and indirect downstream regulation of cellular metabolism (Martinez-Outschoorn et al., 2017). To regulate glucose metabolism, macromolecular biosynthesis, and redox balance, metabolic factors such as growth factors and cytokines activate the PI3K-AKT pathway through a series of biochemical reactions (Zhang et al., 2013). Combination therapy with the mTOR inhibitor sirolimus and daily low-dose cyclophosphamide was administered to 10 patients with advanced unresectable conventional CS. Among them, six patients (60%) had at least 6 months of disease stability, three (30%) had progressive disease, and one (10%) had a partial response (Bernstein-Molho et al., 2012). When CS cell lines are treated with mTOR inhibitors, such as rapamycin or sapanisertib, a continuous decrease in their activity and a decrease in oxidative and glycolytic metabolism are observed, indicating the importance of the mTOR pathway in CS cell metabolism (Slotkin et al., 2015; Addie et al., 2019). Clinical studies targeting mTOR pathway inhibition in CS are likely to provide an effective treatment plan for CS patients in the future.

##### 4.2.2.2 Hedgehog pathway

The Hedgehog (Hh) pathway is a signaling cascade that plays crucial roles in cell differentiation, stem cell maintenance, and cell proliferation (Jia et al., 2015). The Hh pathway exerts powerful metabolic effects by controlling the production, processing, secretion, and transportation of Hh ligands. Additionally, dysregulation of the Hh pathway leads to the development of many cancers, including CS (Tiet et al., 2006). The Hh activates transcriptional processes mediated by the glioma-associated oncogene (GLI) by binding to the transmembrane protein (PTCH1) and smoothened (SMO) receptors. Under Hh ligand deficiency, SMO is inhibited by PTCH1. In the presence of Hh ligands, SMO is released by inhibition, activating SMO-mediated GLI transcription factors, thereby initiating a series of cellular responses (Tiet et al., 2006). In addition, a negative feedback loop regulated by the Indian hedgehog gene and parathyroid hormone-related protein is involved in the pathogenesis of CS. A preclinical study has shown that saridegib (IPI-926), an oral Hh inhibitor, has good therapeutic effects in primary CS xenotransplantation in mice. However, in a double-blind randomized placebo-controlled phase II trial, IPI-926 treatment was terminated because of its unsatisfactory clinical efficacy in advanced CS patients (Campbell et al., 2014). In another single-arm phase II experiment, vismodegib (GDC-0049), an Hh pathway inhibitor for basal cell carcinoma, showed activity in patients with grade 1 or grade 2 traditional CS. However, the clinical trial results were unsatisfactory because the primary endpoint of the six-month clinical benefit was not achieved (Italiano et al., 2013a). Although the clinical trial results are not ideal, more effective Hh pathway inhibitors will be investigated in clinical research and treatment in the future (Wu et al., 2017).

##### 4.2.2.3 Angiogenesis pathway

The angiogenesis pathway can serve as a potential target for the effective treatment of CS. The vascular system provides abundant nutrients for tumor growth and enhances tumor vascularization (Martinez-Outschoorn et al., 2017). The formation of new blood vessels that promote tumor growth, proliferation, and metastasis is inseparable from the expression of vascular endothelial growth factor (VEGF). Studies have reported that the expression of VEGF-A changes with the grading of CS tumors, suggesting that anti-angiogenic therapy can provide a new treatment method for patients with CS (Kalinski et al., 2006). Drugs that affect angiogenesis, such as small-molecule tyrosine kinase inhibitors and human monoclonal antibodies, have emerged in the development of anti-angiogenic therapies. Pazopanib, a multitarget receptor tyrosine kinase inhibitor, inhibits angiogenesis. In xenogenic CS mice, pazopanib significantly affects tumor vascular density, metabolism, and size (Tlemsani et al., 2023). Chow et al. (2020) have conducted a single-arm, prospective, multicenter study to investigate the safety and efficacy of pazopanib in the treatment of unresectable or metastatic traditional CSs. This study included 47 patients who received 800 mg of pazopanib orally once daily for a continuous 28-day cycle until disease progression or unacceptable toxicity occurred. The results show a disease control rate (DCR) of 43% in patients with primary and advanced conventional CSs after 16 weeks of pazopanib treatment. Importantly, the median overall survival (OS) was 17.6 months, which is a significant outcome for patients and provides direct evidence of the positive activity of pazopanib in traditional chemotherapy-resistant diseases.

The latest research indicates that inhibitors targeting some other key signaling pathways have shown potential in the treatment of chondrosarcoma. For example, research on Wnt signaling inhibitors in animal models has shown that they can effectively inhibit tumor growth and metastasis (Liano-Pons et al., 2021). Notch signaling inhibitors have also been shown in preclinical studies to inhibit tumor stem cells and prevent tumor progression (D’Assoro et al., 2022). In addition, combining inhibition of multiple signaling pathways may achieve better results in the treatment of chondrosarcoma. For example, the strategy of jointly inhibiting the Wnt and Notch pathways has shown significant anti-tumor effects in experimental models (Luo et al., 2023). In addition, researchers are exploring the use of RNA interference technology or small molecule compounds to precisely regulate signaling pathways, in order to improve the specificity and effectiveness of treatment (Malakoti et al., 2023).

#### 4.2.3 Immunotherapy for CS

Immunotherapy is an antitumor therapy that targets cancer cells by activating the autoimmune system. The antitumor effects of immune cells can be influenced by an altered metabolic landscape of the tumor microenvironment (Wilde et al., 2017). In recent years, immunotherapy for other types of cancer has been successfully applied; however, research on immunotherapy and the immune microenvironment in CS is still relatively weak. Research has shown that CS is associated with an immune state, and the expression of programmed cell death (PD-1) in CS tissue is higher than that in osteochondroma and normal bone tissue (Torabi et al., 2017). PD-1, an immune checkpoint protein, plays an important role in regulating immune response, promoting immune tolerance, and preventing overactivation (Zhang and Zhang, 2020). When PD-1 is activated by its ligand Programmed Cell Death Ligand 1, it weakens T cell-mediated immune responses through an Interleukin-2-dependent pathway (Chikuma et al., 2009). Paoluzzi et al. have analyzed the efficacy of nivolumab (an anti-PD1 monoclonal antibody) in two patients with CS in a retrospective study conducted in 2016. One patient with mesenchymal CS had stable disease after using nivolumab for four cycles, while another patient with undifferentiated CS showed a partial response after using nivolumab alone for six cycles (Paoluzzi et al., 2016). SARC028 is a phase II clinical trial of two cohorts, a single-arm, multicenter, and open-label trial, to evaluate the role of the anti-PD-1 antibody pembrolizumab in advanced soft tissue sarcoma and osteosarcoma. This study included five patients with CS, and only one patient was found to have stable disease. Among the remaining four patients, one patient had a partial response and three patients had disease progression (Tawbi et al., 2017). In a case report published in 2023, Nowak et al. tracked a woman with a history of stage IV primary CS of the right shoulder who experienced recurrence after radical resection. After receiving pembrolizumab every four cycles, the patient underwent imaging examinations to evaluate the response to treatment. After 23 cycles, the patient achieved significant results, indicating that pembrolizumab has sustained efficacy in controlling the progression of CS (Cohen-Nowak et al., 2023). Therefore, future research directions for the treatment of CS may involve more applications of novel drugs (such as immunotherapy, T-cell therapy, and combination therapy) and metabolic drugs (Micaily et al., 2021).

Currently, multiple clinical trials of immunotherapy for chondrosarcoma are underway. These trials not only evaluate the efficacy of immune checkpoint inhibitors, but also include novel immunotherapy strategies such as tumor vaccines and bispecific antibodies. Preliminary results indicate that combining other treatment methods, such as radiotherapy or chemotherapy, may enhance the effectiveness of immunotherapy. For example, the strategy of combining radio therapy with PD-1 inhibitors has shown synergistic anti-tumor effects in cancers (Chami et al., 2023), suggesting that combination therapy may be an important direction in the future.

#### 4.2.4 Proton therapy for CS

Owing to its deep location and high recurrence rate, skull base CS still faces challenges in treatment despite its low risk of metastasis (Kremenevski et al., 2020). Surgical treatment alone can increase the local recurrence rate; thus, it is difficult to completely remove the tumor surgically. Therefore, radiation therapy is particularly important in the treatment of skull base CSs. The most widely used photon therapy, owing to its inability to balance the actual effective dose and normal tissue tolerance, usually does not achieve the expected clinical results (Gordon et al., 2021). Proton therapy has received increasing attention owing to its advantages in dosimetry compared to photon-based radiation therapy. Proton therapy is utilized with a fixed horizontal proton beam in a seated position, supported by cone-beam computed tomography (CT) and standard mask mobilization (Balakin et al., 2018). Proton therapy utilizes the Bragg peak to deliver a high standard RT dose to a target volume with a steel dose falloff, potentially minimizing the radiation delivered to normal adult structures (Oike et al., 2016; Mercado et al., 2019). Holtzman et al. (2019) have analyzed the efficacy of 43 patients with skull base CS who received dual scattered proton therapy in a univariate outcome study. The results show that with a median follow-up of 3.7 years, at 4 years of local control, the overall survival and toxicity-free survival rates were 89% and 95%, respectively. Radiotherapy-related toxic reactions can be divided into acute (during radiotherapy) and late (after radiotherapy) reactions. During radiotherapy, acute grade 1 and grade 2 toxicity reactions occur to varying degrees in patients receiving proton therapy, leading to symptoms such as radiation dermatitis, hair loss, and mucositis. However, grade 3 acute toxicity associated with radiotherapy has not yet been reported. Furthermore, regarding late-stage toxicity, there were six grade 3 events. Although level 3 toxicity related to radiation was 95% during the four-year follow-up period, the number of adverse events gradually increased after 4 years. In the past decade, proton therapy has made significant progress in the treatment of skull base tumors. Further research is required to explore the application of proton therapy in the treatment of skull base CSs (Holtzman et al., 2019). With the availability of advanced and informative imaging and treatment planning tools, research has begun to quantify the effect of uncertainty on proton therapy (Mohan and Grosshans, 2017).

With the continuous development of proton therapy technology, its application prospects in the treatment of chondrosarcoma are broad. Future research may focus on the following aspects: improve the accuracy of proton beams and further reducing damage to normal tissues through advanced imaging techniques and computer-aided design; study the effects of proton therapy combined with chemotherapy, immunotherapy, and explore new strategies for synergistic therapy; based on the specific situation of the patient and tumor characteristics, develop personalized proton therapy plans to maximize treatment effectiveness.

#### 4.2.5 Chemotherapy of CS

Currently, the efficacy of chemotherapy for CSs is not ideal. An effective chemotherapeutic regimen for patients with advanced CS has not yet been proposed (Zajac et al., 2021). Although the role of chemotherapy in conventional CSs remains unclear, some studies have shown that mesenchymal and dedifferentiated CSs are more sensitive to chemotherapy (Italiano et al., 2013b).

According to current treatment guidelines, local and metastatic dedifferentiated CSs should be treated with the same treatment regimen as osteosarcoma, whereas mesenchymal CS typically requires the same treatment regimen as Ewing’s sarcoma (Rock et al., 2022). In a retrospective analysis of patients with undifferentiated CS published by Maldegem et al., researchers have reported the treatment outcomes of chemotherapy drugs in patients with unresectable undifferentiated CS. The progression-free survival (PFS) of patients treated with doxorubicin monotherapy was 5.5 months, whereas that of patients treated with a combination of doxorubicin and cisplatin was 2.9 months. According to the results, the monotherapy with doxorubicin has better PFS than the combination therapy of doxorubicin and cisplatin (van Maldegem et al., 2019). The combination of surgery, chemotherapy, and radiation therapy for mesenchymal CS has better efficacy, with an initial chemotherapy regimen similar to that for Ewing’s sarcoma and other soft-tissue sarcomas. Therefore, chemotherapy for mesenchymal CS generally involves alternating cycles of etoposide + ifosfamide and doxorubicin + vincristine + cyclophosphamide (Rutkowski et al., 2022). To analyze multidisciplinary treatments, Strach et al. have conducted a multicenter cohort study of 22 patients with mesenchymal CS. They found that 16 of the 22 patients underwent surgical treatment (84%, eight patients underwent surgery only) and 10 patients received systemic chemotherapy (53%, three patients received neoadjuvant chemotherapy, and two patients received adjuvant chemotherapy). The median OS was 104.1 months (95% confidence interval [CI]), and the OS of patients with localized mesenchymal CS who underwent surgical resection of the primary tumor improved (Strach et al., 2023). A retrospective study of 25 patients with unresectable mesenchymal CS showed that a multidrug chemotherapy regimen based on doxorubicin was more advantageous than other chemotherapy regimens based on other drugs, and the PFS was longer (Tsukamoto et al., 2014; Tansir et al., 2020). Although the efficacy of chemotherapy is limited in patients with advanced CS, young patients may consider active local treatment combining chemotherapy with surgery or radiotherapy, whereas palliative treatment is more suitable for older patients in advanced stages (Dudzisz-Sledz et al., 2023).

Researchers are exploring new chemotherapy drugs and combination therapies to address the issue of drug resistance in chondrosarcoma. For example, the use of novel DNA repair inhibitors or targeted drugs combined with traditional chemotherapy drugs aims to enhance the sensitivity of tumor cells to chemotherapy. In addition, research on nanomedicine carriers has also provided new directions for chemotherapy of chondrosarcoma. By delivering chemotherapy drugs through nanoparticles, the accumulation of drugs in tumor tissues can be increased (Alexiou et al., 2023), and the toxicity to normal tissues can be reduced, thereby improving the therapeutic effect.

#### 4.2.6 Surgical treatment for CS

Surgical resection remains the most effective method for treating primary or secondary CSs. Surgery for pelvic CS is complex and challenging and faces challenges such as high surgical difficulty and trouble in functional reconstruction (Ji et al., 2013). Reconstruction methods for pelvic defects can be divided into non-prosthetic and prosthetic reconstructions. Non-prosthetic reconstruction methods include the Harrington method (Kask et al., 2020), autologous bone transplantation (Lin et al., 2018), inactivated replantation, and allogeneic bone transplantation (Karim et al., 2015); however, there are problems with these methods such as bone non-healing and multiple complications. Prosthetic reconstruction methods include saddle-type prostheses (Danisman et al., 2016), ice cream stem/iliac stem prostheses (Issa et al., 2018), combination-type semi-pelvic prostheses, and customized semi-pelvic prostheses (Wang et al., 2019). The first two require high iliac bone preservation and are associated with a high risk of loosening. Combination-type prostheses also have problems, such as poor interface matching and mechanical failure of the prosthesis connection (Abdel et al., 2017). With continuous progress in technology, new treatment methods and technologies have provided novel opportunities to solve these problems. In recent years, the emergence of 3D printing technology has brought tremendous changes and developments in the surgical treatment of pelvic CS (Traub et al., 2013; Chan et al., 2016).

The application of 3D printing technology has led to significant progress in the surgical treatment of pelvic CS, and the continuous development of its manufacturing materials and technology has provided better choices for pelvic CS surgery. Polymers and metals are commonly used in the manufacturing of materials. Polymer implants have good biocompatibility and plasticity and can be customized according to individual patient differences. In contrast, metal implants have a higher mechanical strength and biological stability, making them suitable for situations with high load-bearing requirements. In addition, new materials, such as ceramics and bioactive materials, are constantly being explored and applied (Aimar et al., 2019).

Regarding manufacturing technology, 3D printing provides feasible solutions for manufacturing prostheses. The three most commonly used 3D printing technologies are selective laser melting, selective laser sintering, and electron beam melting. Prostheses can be manufactured through layer-by-layer stacking, based on a patient’s CT or MRI image data. Compared to traditional manual production, 3D printing technology can not only improve the accuracy and adaptability of the prosthesis but also reduce surgical time and alleviate postoperative pain in patients (Wong et al., 2015).

Accurate reconstruction of the hip joint and pelvis is crucial for the functional recovery of patients undergoing surgery for pelvic CSs (Bus et al., 2017). 3D-printed prostheses can be accurately positioned and customized according to the patient’s pelvic structure and osteotomy position. Before surgery, the simulation design of the prosthesis can be conducted by introducing imaging data, such as CT and MRI of the patient, and necessary adjustments can be made to the prosthesis to ensure the reconstruction effect after the removal of pelvic CS (Wang et al., 2015). 3D-printed prostheses can ensure the accuracy and safety of functional reconstruction during surgery, and the bone growth interface thereof can be organically combined with the patient’s bone tissue, providing better stability and functional reconstruction effects.

However, 3D-printed prostheses also have limitations in the surgical treatment of pelvic CSs. At present, there are still certain shortcomings in manufacturing materials and technologies, such as durability, wear resistance, and corrosion resistance, which require further optimization. 3D-printed prostheses’ high manufacturing costs and long manufacturing cycles limit their popularity and application in clinical practice (Bus et al., 2014). Additionally, the long-term safety and reliability of 3D-printed prostheses also require further evaluation and verification.

### 4.3 Limitations

Although this study used bibliometric and visual analysis methods to analyze the current status and trends in CS research, the CiteSpace and VOSviewer software cannot completely replace systematic retrieval and, therefore, have some limitations. First, we only selected literature from the WoS database as the data source, and the selected literature was not sufficiently comprehensive. In addition, the literature we collected was from 2003 to 2022; however, with the continuous updating of literature in the WoS database, there may be a slight difference between the search results of this study and the actual number of included studies. Finally, several core keywords in this article were not fully included in the system analysis, which may have been affected by incomplete keyword extraction.

## 5 Conclusion

We searched and analyzed 4,149 articles in the field of CS research published between 2003 and 2022. Despite some limitations, our study indicates that publications related to CS research are increasing rapidly worldwide. Using the CiteSpace and VOSviewer software for visual analysis, we found that research in the field of CS is increasing every year. Globally, the United States and China are at the forefront of CS research. Furthermore, over the past two decades, the global trend in CS research has focused primarily on clinical studies with basic research as a supplement. Therefore, future studies should focus on emerging hotspots related to CS treatment, such as proton therapy, radiotherapy, chemotherapy, and immunotherapy. Simultaneously, to promote the widespread application of 3D printed prostheses in the surgical treatment of pelvic CS, future research should focus on improving materials, technology, and manufacturing efficiency, as well as reducing costs and strengthening the evaluation of its long-term safety and reliability.
